# Knowledge and Role of Paediatricians/Paediatric Residents in Infant Oral Healthcare and Dental Home across Saudi Arabia: A Cross-Sectional Study

**DOI:** 10.3390/children10091579

**Published:** 2023-09-21

**Authors:** Satish Vishwanathaiah, Prabhadevi C. Maganur, Dhalia Hassan Albar, Ranya Hassan Albar, Mohammed Abdurabu Jafer, Safeyah A. Baeshen, Imtinan Ahmed Madkhali, Enas Jaber Mohana, Jawaher Saleh Sahli, Alhanouf K. Alnajdi, Manal Kinani Tahhah, Varsha Manoharan

**Affiliations:** 1 Division of Pediatric Dentistry, Department of Preventive Dental Sciences, College of Dentistry, Jazan University, Jazan 45142, Saudi Arabia; svishwanathaiah@jazanu.edu.sa (S.V.); dhalia.h.albar2023@student.riyadh.edu.sa (D.H.A.); 2 Preventive Dentistry Department, Pediatric Dentistry Division, Riyadh Elm University, Riyadh 12734, Saudi Arabia; 3 Independent Researcher, Jeddah 45142, Saudi Arabia; rania.albar@hotmail.com; 4 Division of Public Health, Department of Preventive Dental Sciences, College of Dentistry, Jazan University, Jazan 45142, Saudi Arabia; majafar@jazanu.edu.sa; 5 College of Dentistry, Jazan University, Jazan 45142, Saudi Arabia; 201703429@stu.jazanu.edu.sa (S.A.B.); 201702027@stu.jazanu.edu.sa (E.J.M.); 201705551@stu.jazanu.edu.sa (J.S.S.); 201601652@stu.jazanu.edu.sa (A.K.A.); 201701405@stu.jazanu.edu.sa (M.K.T.); 6 Department of Public Health Dentistry, KVG Dental College and Hospital, Sullia 574327, India; varsha.m.nair91@gmail.com

**Keywords:** dental home, infant oral healthcare, paediatricians, paediatric residents

## Abstract

The prevention of oral diseases in children is highly achievable through providing early exposure to oral healthcare habits, which would make children more receptive towards dental services. A cross-sectional study used a structured, self-explanatory questionnaire to evaluate the knowledge and role of 190 paediatricians and paediatric residents towards infant oral healthcare and the dental home across Saudi Arabia. The authors sent a link to the questionnaire, comprising 36 questions, by email, as a Google e-form. The response rate obtained was 87.36%. A major proportion (95.8%) of the respondents considered that paediatricians play an important role in promoting oral health. Around 45.8% of the practitioners recommended tooth brushing after the eruption of the first tooth. About 38% of the practitioners were unaware of the children’s first dental visit. The majority (95.2%) agreed that there should be an increased awareness regarding home dental habits. Most of them (78.3%) examined the oral cavity for dental problems as a part of routine childcare, and the majority (75.3%) admitted that they did not evaluate fluoride needs. A statistically significant higher mean knowledge score was observed for practitioners with more than 30 years of experience (9.35 ± 2.29), and there was a significant positive correlation (r = 0.486) between the knowledge score and mean score for the role of paediatric practitioners. Overall, the study concluded that the knowledge of paediatricians and paediatric residents in SA towards infant oral healthcare and the dental home was inadequate, and their role in maintaining oral health was found to be moderately satisfactory.

## 1. Introduction

Dental caries is the most widespread oral affliction affecting the young population. This disease has the potential to stir up numerous unpleasant conditions in the oral cavity and throughout the body [1]. In toddlers and infants, dental caries adopts a unique pattern. An array of names and terminologies has emerged to pinpoint the presence of dental caries among this group. Early definitions were centered on the root cause, emphasizing the incorrect nursing practices responsible for their emergence [2,3]. 

“Early childhood caries” (ECCs) is gaining traction among dental professionals and researchers in the field. Early childhood caries, along with its more severe variant, initiates soon after tooth eruption, manifests on smooth surfaces, advances swiftly, and leaves a long-lasting, negative impact on dental health [4,5], which can impede a child’s growth, lead to considerable discomfort, and reduce their overall quality of life [5]. In 1986, the American Academy of Pediatric Dentistry (AAPD) introduced the first infant oral health-care policy statement approach [6]. This policy has now been in place for more than 37 years. 

Infant oral healthcare is the fundamental cornerstone of establishing healthy oral habits from infancy and beyond, to achieve optimal dental health [7]. An infant oral healthcare program has the potential to detect, halt, and improve detrimental parenting methods that may jeopardize a child’s dental health [8,9]. The patient-centered medical home (PCMH) is a revolutionary approach to primary care, designed to elevate the quality of healthcare and promote seamless integration [10]. The patient-centered dental home (PCDH) enters the playing field as the dental equivalent of the PCMH, paving the way for enhanced medical–dental integration. To ensure optimal oral health, experts suggest establishing a PCDH early on, ideally by the age of one, as per the AAPD guidelines [9,11]. As we propel forward into the realm of caries risk detection and preventative measures, the dental home can be the prime destination to forge and execute scientific and evidence-based strategies for efficient disease management [12].

Early screening for children below the age of one presents an excellent opportunity to identify risk factors. It is imperative to shift from the conventional surgical approach in dentistry towards preventive oral measures that commence in the prenatal or infancy phase [13]. Children under three are not frequently seen by dentists, which leaves them vulnerable to oral diseases. Well baby visit programmes provide paediatricians with the opportunity to examine newborns from birth, thereby offering the optimal means for the timely detection of dental issues. By educating parents on oral preventive healthcare, paediatricians are better positioned to provide screening services for early diagnosis and to make referrals where and when needed [2,13]. 

In a recent study conducted in Saudi Arabia (SA), the proportion of dental caries among primary teeth ranged from 0.21 to 1.00, painting a troubling picture [1]. The paucity of precise data on the extent of knowledge among paediatricians in Saudi Arabia regarding the prevention of dental caries in children beckoned us to embark on this study. Hence, our study was undertaken to delve into the knowledge and role of paediatricians/paediatric residents towards infant oral healthcare and the dental home across Saudi Arabia.

## 2. Materials and Methods

### 2.1. Study Design and Study Setting

This cross-sectional study was conducted among paediatricians and paediatric residents practicing or studying in various places in Saudi Arabia. A self-administered closed-ended structured questionnaire was framed and distributed via email as a Google e-form, and the overall data collection lasted for three months (from September to November 2022).

### 2.2. Sample Size 

The sample size was obtained from the following parameters derived from a previous publication [14]. With a 95% confidence interval, a precision of 0.04, and the proportion of paediatricians assessing dental problems during routine physical examinations being 92.6% in the United States (US), the minimum sample size required to reject the null hypothesis was estimated at 171. Ten percent of the estimated sample size was added to compensate for sampling loss, if any, and, thus, the final sample size was a total of 190 participants.

### 2.3. Selection Criteria

Paediatricians and paediatric residents who were willing to give informed consent for participation through Google e-forms were included in the study. Those who did not respond, even after being sent three reminders with a two-day interval in between, were excluded.

### 2.4. Data Collection Process and Variables Assessed

The questionnaire comprised three sections. The first part consisted of items to assess demographic data; the second and third part comprised questions to assess the knowledge and role of paediatricians and paediatric residents towards infant oral healthcare and the dental home.

A set of 36 questions in English was formulated based on a literature review [2,7,15]. The content validation of the questionnaire was performed by experts. Each question was assessed for its relevance via calculating Aiken’s Index [16]. Questions that obtained a score of ≥0.6 were included in the proforma. The reliability of the questionnaire was assessed via Cronbach’s α value, which ranged between 0.75 and 0.87, with a median of 0.81, showing good reliability. A link to the questionnaire comprising 33 questions was sent via a Google e-form to 190 paediatricians and paediatric residents, and each participant was allowed to respond only once. Respondents were automatically directed to the study details after informed consent was obtained, and they were allowed to respond only once. Every piece of information collected was checked for completeness and consistency. In order to calculate the mean scores for knowledge and the mean scores for the role of paediatric practitioners, a score of 1 was awarded for the correct responses to each question (except for question 19 in Table 1), and all other incorrect answers were assigned a score of 0.

### 2.5. Statistical Analysis

To analyze the descriptive statistics of the practitioner’s response to various questions, we utilized IBM SPSS (Statistical Package for Social Sciences) Version 21.0, Chicago. ANOVA was used to assess the variation in mean scores for knowledge and mean scores for the role of paediatric practitioners based on their years of experience. The Pearson correlation was used to find the relationship between the mean scores for knowledge and the mean scores for the role of paediatric practitioners. The level of significance was set at a *p* value < 0.05.

### 2.6. Ethical Considerations

The current study was carried out in accordance with the Declaration of Helsinki 1964 and was approved by the standing committee for scientific reseaech, the Jazan University Review Board at Jazan University (HAPO-10-Z-001), reference no REC-44/04/378.

## 3. Results

Out of the 190 Google e-forms distributed, 166 were returned completed, yielding a response rate of 87.36%. The analysis of the demographic data revealed that most of the practitioners were female (69.88%) paediatricians (74.7%) with experience of less than 5 years (25.9%), as shown in Table 1.

On investigating the knowledge part, we found that most respondents (77.7%) agreed that both paediatricians and paediatric dentists were responsible for infant oral healthcare. A major proportion (95.8%) of them considered that paediatricians play an important role in promoting oral health, and a vast majority (95.8%) agreed that milk teeth were very important. Around 39.7% of the practitioners strictly did not recommend bottle feeding for children. More than half of the practitioners (51.2%) agreed that they observed early childhood caries in OPD at least once a week. A vast majority (99.4%) of them stated that dental caries assessment and counselling on the prevention of dental caries (98.8%) should be a part of routine childcare. Around 45.8% of practitioners recommended tooth brushing after the eruption of the first tooth. A large proportion of practitioners willingly (97%) referred parents to a general dentist (55.9%) when dental problems were seen in children. About 38% of the practitioners were unaware regarding children’s first dental visit. Most (80.1%) of the practitioners had not attended any dental educational program on oral health in children. The majority (95.2%) felt the need for a dental educational program on oral health in children and to publish preventive dentistry articles in medical journals, too. Most of them (90.4%) agreed to establish a dental home, along with a medical home. Around 98.2% of them considered proper oral health an integral part of general wellbeing. About 76.5% of the practitioners discussed oral health problems with the parents, and most of them (98.2%) believed that feeding habits played a major role in creating dental problems. More than half (59%) of the practitioners agreed that there was not a dental home in their hospital, and two thirds (64.7%) of them agreed that they undoubtedly referred infants to dental home if they had dental problems. It was agreed by 34.9% of the practitioners that dental caries assessment was the major procedure performed in the dental home. More than half (51.8%) of the participants agreed that prenatal counselling is a part of the dental home and that the paediatric postgraduate curriculum promotes oral healthcare. The majority (95.2%) agreed that there should be an increased awareness regarding the dental home, as depicted in Table 2.

Regarding the role of paediatric practitioners, the majority (96.4%) of them reported that they treated children aged 0–36 months [0–3 years], and two thirds (69.3%) evaluated their oral health, too. Most of them (78.3%) examined their oral cavities for dental problems as part of routine childcare. The majority (93.4%) agreed that they provided nutritional counselling, and 74.83% of them had given nutritional counselling particularly for dental caries. Bottle feeding was never recommended by 39.7% of practitioners. Most of them (77.7%) discussed baby bottle decay risk with parents. Around 72.3% of the practitioners educated parents about dental decay and about the prevention of tooth decay (68.7%). The majority (75.3%) admitted that they did not evaluate fluoride needs. A major proportion of practitioners (81.9%) agreed that they provided counselling on the importance of tooth brushing and had given oral hygiene instructions (69.3%). Sadly, practitioners confirmed that they had talked to parents about their first dental visit only occasionally (50.6%), as described in Table 3.

A statistically significant difference was found for the mean knowledge score in relation to years of experience among paediatric practitioners. The mean knowledge score was found to be higher in practitioners with more than 30 years of experience (9.35 ± 2.29). There was no statistically significant difference observed for the mean score for the role of paediatric practitioners based on years of experience, as shown in Table 4 and Table 5.

A significant positive correlation (r = 0.486) was observed between the knowledge score and mean score for the role of paediatric practitioners, as depicted in Table 6.

## 4. Discussion

The contribution of the non-dental workforce to the improvement of oral health outcomes in children is becoming an increasingly important concern on a global scale [17]. Oral health is considered an essential subject of collaboration between healthcare professionals within primary healthcare settings, according to the World Health Organization (WHO) [18]. It has been recognized for more than a decade now that doctors occupy a critical position in the promotion of oral health among children. Paediatricians have a role that is particularly important in this regard [19], despite the fact that advocating for oral health is a job that is shared by a number of different healthcare professions.

Medical and dental professionals must work together to provide comprehensive childcare and age-appropriate treatments [2]. Paediatricians and family physicians treat children from infancy to adolescence. To achieve child oral health goals, paediatricians must recognize early dental disorders, promote preventive practices, and make appropriate referrals [20]. This can ensure infant oral health and establish a dental home to promote preventive education and dental care, leading to a lifetime of protection against avoidable oral diseases, maintaining a healthy relationship between patients and dentists, and creating a warm and non-intimidating environment for infants and toddlers in dental hospitals [4,13]. However, doubts remain concerning early oral healthcare, dental referrals, and paediatricians’ preventive oral healthcare measures. This study is the first to assess Saudi Arabian paediatricians and paediatric residents’ knowledge and role in newborn oral healthcare and the dental home.

Our research emphasized that paediatricians had a strong conviction in their ability to promote good oral health practices among children, which was consistent with the findings of Lewis et al. [14], Chouchene et al. [21], and Prakash et al. [22]. These studies reported that most of the paediatricians regularly examined their patients’ oral cavities and provided guidance on oral health during well childcare visits. Contrary to these findings, Shetty and Dixit [23] reported that less than half of paediatricians in North Karnataka had conducted routine oral check-ups. In another study conducted by Gupta et al. [24], over 80% of paediatricians recognized the importance of primary dentition, which was similar to our study’s outcomes. Most of the paediatricians in our study had provided nutritional counselling, as well as counselling regarding the prevention of dental caries, which was in accordance with other previous studies conducted in the US and India [14,25], in which most participants had warned on the importance of preventing dental problems. It is said that children typically have 11 well visits with a physician by the age of thre. Therefore, incorporating dietary counselling into these visits can be beneficial in preventing ECC by providing parents with guidance on the impact of improper feeding practices and the consumption of sweetened drinks at night [26].

In this study, we found that most paediatricians advised patients with oral concerns to consult a dentist, which was in line with a Canadian paediatrician study [22], in which it was most commonly suggested that tooth decay be treated by a dentist. Sezer et al. [27] found a link between paediatricians’ expertise and referral rates, showing that more knowledgeable practitioners were more likely to recommend a dentist visit.

It was intriguing to observe the wide range of views among paediatricians regarding the optimal age for the first dental check-up and the significance of the initial dental examination after birth. This variability could stem from the fact that some medical professionals are not up to date with the AAPD recommendations for preventative dental care in children. As cited by Karasz [28], most paediatricians are aware of the referral protocols for dental visits before the age of 12 months; however, few actually execute the recommendation. Many studies have exposed a lack of awareness regarding dental referrals for young children [25,29,30,31], and it is evident that several paediatric practitioners in this research were also not aware of when the child’s first dental visit should be scheduled. According to the AAPD guidelines, a child’s initial dental examination should occur within six months of the emergence of their first set of teeth, or by their first birthday [15,24].

In a study by Chouchene F et al. [21], it was found that only 32% of paediatricians suggested a first dental check-up for their patients between the ages of six months and one year. This finding agrees with our research, in which 32.5% of respondents recommended a dentist visit after the child’s first primary teeth had erupted. Interestingly, a similar study in the US showed that a meager 17% of paediatricians advocated for a first dental check-up before the age of one. Shockingly, 50% of them believed that the first dental appointment should not occur before three years of age [14]. Similarly, a survey conducted by Brickhouse et al. [15] in Virginia reported that only 5% of paediatricians suggested that a child should have had a dental check-up by the age of one.

Shetty and Dixit [23] conducted research that recommended that tooth brushing should start only after 18 months, and weaning should occur between 12 and 24 months of age. However, according to the AAPD, brushing should commence as soon as the first tooth appears in the oral cavity [32]. Sharma et al. found that the initiation of oral hygiene practices before and during the first tooth eruption was important but was less commonly implemented by paediatricians [33]. Our study also reflected similar results; only 45.8% of paediatricians recommended tooth brushing after the first tooth eruption. The surveyed paediatricians in this study were unaware of the general AAPD recommendations. More than one third of the paediatricians who took part in our study demonstrated adequate knowledge regarding bottle feeding practices and the negative impact they can have on a child’s dentition. Research has shown a correlation between a higher prevalence of dental caries in children who are bottle-fed at night [34]. This finding aligns with a survey conducted in Chandigarh, which showed a good proportion of paediatricians having knowledge about the harmful effects of bottle feeding [35]. 

Unfortunately, many paediatricians in our research did not assess the need for fluoride supplementation, unlike the study by Lewis and colleagues [14], in which 94% of respondents were confident in determining the fluoride supplementation dose. Kalkani and colleagues found that just one in five UK paediatric postgraduate trainees could correctly identify fluoride supplement doses [36]. Shetty and Dixit [23] found that 92% of paediatricians were aware of fluoride as a prophylactic intervention but did not specify how many recommended it. Another study report from India [35] revealed that around two thirds of paediatricians were aware of the caries-protective role of fluorides.

In a study by Goyal A et al. [35], it was found that only 24.5% of paediatricians referred children with special healthcare needs to dentists for treatment, indicating a lack of appropriate referrals. However, in our research, a good percentage of practitioners agreed that they made appropriate referrals whenever dental problems were seen. A Turkish study [27] showed that only 10.8% of participants received oral health education during their residency or at medical school. Additionally, paediatricians have voiced their concerns over the inadequate amount of time spent on oral health education at the undergraduate, and graduate, and continuing medical education levels. Conversely, more than half of the participants in our research agreed that the current postgraduate paediatric curriculum promoted oral healthcare.

Balaban et al. [37] reported that 83.4% of paediatricians in Brazil classified the oral health content in their medical education as either non-existent or deficient. There are various studies [23,38,39,40] published in the literature that have recommended further training to improve paediatricians’ confidence and knowledge of dental topics. Based on our observations, it seems that most participants expressed the desire for a dental education program that focuses on the oral health of children.

Results from another survey developed by Vaidya et al. [41] showed that, although most of the paediatric residents regularly encountered dental problems and are involved in their prevention, only half of the paediatric residents were aware of the existence of a dental home in their hospital. Similarly, in our study, less than half of the practitioners reported the presence of a dental home in their hospital, but 64.7% stated that they would refer infants to dental homes whenever they came across any dental issues. Many practitioners demonstrated an incomplete knowledge about the procedures performed in a dental home, which was similar to the findings of Lewis and colleagues [14].

In another study [41], it was reported that 50% of the paediatric residents in India were aware that prenatal counselling is an essential aspect of the dental home, which was in accordance with our observations, which showed 51.8% of practitioners being aware of the same. The paediatric residents were found to be more involved in procedures such as the child’s development, diet counselling, and the diagnosis of dental caries compared to immunization, trauma cases, and topical fluoride application [41], which was inconsistent with our findings, in which one third of practitioners believed that the major procedure performed in the dental home was dental caries assessment, followed by the child’s growth and development and diet counselling. In general, the results of our study exhibited a paucity of awareness about the concept of a dental home.

Another noteworthy finding of our study was that practitioners who had more than 30 years of practice reported significantly higher mean knowledge scores compared to their counterparts, suggesting that practitioners with more experience had more knowledge. A significant positive correlation was also observed between the knowledge score and the mean score for the role of paediatric practitioners. Those who exhibited a higher knowledge played a better role in infant oral healthcare and the dental home. Concurrent observations were observed in a previous study reported by Indira and colleagues [7], in which paediatricians who had more than 10 years of experience showed greater mean knowledge scores.

The results of the current study are noteworthy, yet they should be interpreted with caution, considering their methodological strengths and limitations. Although the data were collected using validated, tools, it is important to consider that this study was conducted solely in Saudi Arabia, which brings into question the generalizability of the results. Secondly, we are not neglecting the possibility of responder bias and the overestimation of the results, which are inherent to any survey. Moreover, we have not made an attempt to assess the barriers to educating paediatricians on children’s oral health. Apart from these limitations, this study identified necessary areas for potential training for paediatricians and revealed the prevailing gap between general and oral healthcare—a disparity that, if bridged, could foster healthier and enhanced lifestyles for children.

We would recommend conducting future studies to gather data from diverse populations to bolster the robustness of the conclusions drawn. It is believed that providing a brief training session for paediatricians regarding infant oral health could help in accurately identifying children with oral diseases in a timely manner. Paediatricians can expand their services by conducting basic dental screenings and encouraging parents to establish dental homes for their children. To improve their understanding of oral health, paediatric post-graduate curricula and hospital training should include mandatory dental postings to teach the etiology, symptoms, and preventive strategies of oral diseases. 

## 5. Conclusions

To conclude, the knowledge of paediatricians and paediatric residents in Saudi Arabia towards infant oral healthcare and the dental home was inadequate, and their role in maintaining oral health was found to be moderately satisfactory. Paediatric practitioners who had more years of practice displayed a greater knowledge regarding infant oral health and the dental home. Moreover, there was a significant positive correlation between the knowledge score and mean score for the role of paediatric practitioners. There is a call for more education and training among paediatricians in the area of infant oral health and in conducting oral assessments. Similarly, general dentists require more training on paediatric dental care, specifically relating to infants. Overall, the medical and dental communities need to raise awareness among the public about the importance of early dental visits and of establishing a dental home to prevent early childhood caries.

## Figures and Tables

**Table 1:** The demographic data of the study participants.

SI. No	Parameters	N (%)
1	Gender	
	Male	50 (30.12)
	Female	116 (69.88)
2	Number of years in practice	
	10–20 years	39 (23.49)
	20–30 years	23 (13.86)
	5–10 years	35 (21.08)
	Less than 5 years	43 (25.9)
	More than 30 years	26 (15.66)
5	Occupation	
	Paediatrician	124 (74.7)
	Paediatric Resident	187 (25.3)

**Table 2:** Knowledge of paediatricians/paediatric residents towards infant oral healthcare and the dental home.

SI. No	Questions	Responses	n	(%)
1.	Who is responsible for infant oral healthcare?	Paediatrician	19	11.4
		Paedodontist	18	10.8
		Both	129	77.7
2.	Do you think paediatricians should have role in promoting oral health?	No	7	4.2
		Yes	159	95.8
3.	Do you feel milk teeth are important?	No	7	4.2
		Yes	159	95.8
4.	What is the frequency of early childhood caries in OPD?	At least once a week	85	51.2
		More than once a week	52	31.3
		Never	29	17.5
5.	Do you feel the assessment of dental caries should be a part of routine well child care?	No	1	0.6
		Yes	165	99.4
6.	Do you feel counselling on the prevention of dental caries should be a part of routine well child care?	No	2	1.2
		Yes	164	98.8
7.	What age do you recommend for tooth brushing?	After eruption of all teeth	22	13.3
		After eruption of first tooth	76	45.8
		After a few teeth erupt	68	41.0
8.	Do you refer parents to a dentist when dental problems are seen in children? If yes, to whom do you refer:	No	5	3.0
		Yes	161	97.0
		General dentist	90	55.9
		Paedodontist	71	44.09
9.	Paediatrician awareness about first dental visit:	After eruption of all primary/deciduous teeth	35	21.1
		After eruption of first primary/deciduous tooth	54	32.5
		After eruption of permanent tooth	4	2.4
		At birth	10	6.0
		Do not know	63	38.0
10.	Have you attended any dental educational program on oral health in children?	No	133	80.1
		Yes	33	19.9
11.	Do you feel the need for a dental educational program on oral health in children?	No	8	4.8
		Yes	158	95.2
12.	Do you feel the need to publish preventive dentistry articles in medical journals?	No	8	4.8
		Yes	158	95.2
13.	Do you agree to the establishment of the dental home along with that of the medical home?	No	16	9.6
		Yes	150	90.4
14.	Do you think proper oral health is an integral part of general wellbeing?	No	3	1.8
		Yes	163	98.2
15.	Do you discuss oral health problems with the parents?	No	39	23.5
		Yes	127	76.5
16.	Do you think that there is a role played by feeding habits in dental problems?	No	3	1.8
		Yes	163	98.2
17.	Is there a dental home in your institution/hospital? If yes, do you refer the infant to the dental home if the infant has dental problems?	No	98	59.0
		Yes	68	41.0
		No	24	35.2
		Yes	44	64.7
18.	Is prenatal counselling a part of the dental home?	No	80	48.2
		Yes	86	51.8
19.	Tick the procedures that you think are performed in a dental home	Dental caries assessment	58	34.9
		Growth and development	40	24.09
		Diet counselling	18	10.84
		Topical fluoride application	15	9.03
		Immunization	13	7.8
		Acute cases of trauma	2	1.2
		All of the above	20	12.04
20.	Do you think there should be increased awareness regarding the dental home?	No	8	4.8
		Yes	158	95.2
21.	Does the current postgraduate curriculum in paediatrics promote oral healthcare?	No	80	48.2
		Yes	86	51.8

**Table 3:** The role of paediatricians/paediatric residents towards infant oral healthcare and the dental home.

SI. No	Questions	Responses	N	(%)
1.	Do you treat children aged 0–36 months [0–3 years]?	No	6	3.6
		Yes	160	96.4
2.	Do you evaluate proper oral development?	No	51	30.7
		Yes	115	69.3
3.	Do you examine the oral cavity for dental problems as a part of routine childcare?	No	36	21.7
		Yes	130	78.3
4.	Do you give nutritional counselling? If yes, do you give nutritional counselling (particularly regarding dental caries)?	No	11	6.6
		Yes	155	93.4
		No	46	29.67
		Yes	116	74.83
5.	What will be the time duration suggested to continue bottle feeding?	1 year	60	38.0
		2 years	40	24.1
		Never recommended	66	39.7
6.	Do you discuss baby bottle decay risk with parents?	No	37	22.3
		Yes	129	77.7
7.	Do you educate parents about dental decay?	No	46	27.7
		Yes	120	72.3
8.	Do you educate parents about the prevention of tooth decay?	No	52	31.3
		Yes	114	68.7
9.	Do you evaluate fluoride needs?	No	125	75.3
		Yes	41	24.7
10.	Do you counsel on the importance of tooth brushing?	No	30	18.1
		Yes	136	81.9
11.	Do you give oral hygiene instructions?	No	51	30.7
		Yes	115	69.3
12.	What is the frequency of talking to parents about their first dental visit?	Most of the times	36	21.7
		Never	46	27.7
		Sometimes	84	50.6

**Table 4:** Distribution of the mean knowledge score of paediatric practitioners based on years of experience.

Experience (Years)	Frequency (n)	Mean ± SD	*p* Value
Less than 5 years	43 (25.9)	7.2 ± 2.79	<0.001 *
>5–10 years	35 (21.08)	7.91 ± 2.7	
>10–20 years	39 (23.49)	9.19 ± 1.74	
>20–30 years	23 (13.86)	8.82 ± 2.22	
More than 30 years	26 (15.66)	9.35 ± 2.29	

* denotes statistical significance.

**Table 5:** Distribution of the mean score for role of paediatric practitioners based on years of experience.

Experience (Years)	Frequency (n)	Mean ± SD	*p* Value
Less than 5 years	43 (25.9)	15.32 ± 2.77	0.312
>5–10 years	35 (21.08)	15.82 ± 2.57	
>10–20 years	39 (23.49)	16.35 ± 2.05	
>20–30 years	23 (13.86)	15.17 ± 2.53	
More than 30 years	26 (15.66)	15.61 ± 2.49	

**Table 6:** The correlation between the mean knowledge score and mean score for the role of paediatric practitioners.

Variable	Pearson’s Correlation Coefficient (r)	*p* Value
Mean knowledge score vs. mean score for role	0.486	<0.001 *

* denotes statistical significance.

## Data Availability

Data available on personal request.
